# Decoding Cytokine Dynamics: Wharton’s Jelly Stromal Cells and Chondro-Differentiates in PHA-Stimulated Co-Culture

**DOI:** 10.3390/cells14030174

**Published:** 2025-01-23

**Authors:** Raja Sundari Meenakshi Sundaram, Secunda Rupert, Prasanna Srinivasan, Jeswanth Sathyanesan, Kavitha Govarthanan, Naveen Jeyaraman, Swaminathan Ramasubramanian, Madhan Jeyaraman, Ho Yun Chung, Prakash Gangadaran, Byeong-Cheol Ahn

**Affiliations:** 1 Department of Regenerative Medicine and Research, Government Stanley Hospital, Chennai 600001, Tamil Nadu, India; bt.raji@gmail.com (R.S.M.S.); drsecunda@gmail.com (S.R.); prasannasrinivasan81@yahoo.com (P.S.); 2 Department of Biotechnology, Indian Institute of Technology Madras, Chennai 600036, Tamil Nadu, India; govarthanan_kavitha@yahoo.co.in; 3 Department of Orthopaedics, ACS Medical College and Hospital, Dr. MGR Educational and Research Institute, Chennai 600017, Tamil Nadu, India; naveenjeyaraman@yahoo.com (N.J.); madhanjeyaraman@gmail.com (M.J.); 4 Department of Regenerative Medicine, Mother Cell Regenerative Centre, Tiruchirappalli 620017, Tamil Nadu, India; swaminathan.ramasubramanian@outlook.com; 5 Department of Plastic and Reconstructive Surgery, CMRI, School of Medicine, Kyungpook National University, Kyungpook National University Hospital, Daegu 41944, Republic of Korea; hy-chung@knu.ac.kr; 6 BK21 FOUR KNU Convergence Educational Program of Biomedical Sciences for Creative Future Talents, Department of Biomedical Sciences, School of Medicine, Kyungpook National University, Daegu 41944, Republic of Korea; 7 Department of Nuclear Medicine, School of Medicine, Kyungpook National University, Daegu 41944, Republic of Korea; 8 Cardiovascular Research Institute, Kyungpook National University, Daegu 41944, Republic of Korea

**Keywords:** mesenchymal stromal cells, umbilical cord, chondro-differentiation, cytokines, PHA stimulation, immunomodulation, osteoarthritis

## Abstract

Introduction: Articular cartilage damage presents a significant clinical challenge, with limited options for effective regeneration. Mesenchymal stromal cells (MSCs) derived from Wharton’s jelly (WJ) are a promising cell source for cartilage repair due to their regenerative and immunomodulatory properties. While undifferentiated MSCs have demonstrated potent immunoregulatory effects, the immunomodulatory potential of chondrocytes derived from WJ-MSCs remains underexplored, particularly under inflammatory conditions. This study investigates the differential cytokine expression profiles of WJ-MSC-derived chondrocytes and undifferentiated MSCs under inflammatory stimulation with phytohemagglutinin (PHA) to understand their immunomodulatory capacities. Materials and Methods: WJ-MSCs were differentiated into chondrocytes using a micromass culture system. Differentiated chondrocytes were then co-cultured with immune cells under PHA-induced inflammatory conditions. Control groups included co-cultured cells without PHA activation and chondrocytes activated with PHA in the absence of immune cell interaction. Cytokine expression profiles were analyzed using the RT^2^ Customized Gene Array to evaluate pro- and anti-inflammatory markers. Morphological changes were assessed microscopically. The immunomodulatory responses of chondrocytes were compared to those of undifferentiated MSCs under the same experimental conditions. Results: Chondrocytes co-cultured with immune cells under PHA activation exhibited downregulation of IDO, HLA-G, PDGF, IL-10, TNF-α, IL-6, and IFN-γ compared to undifferentiated MSCs in similar conditions. In non-PHA co-cultured conditions, chondrocytes showed increased expression of IL-6, IFN-γ, IL-4, VEGF, iNOS, PDGF, PTGS-2 and TGF-β, while TNF-α, IL-10, IDO and HLA-G were decreased. In contrast, chondrocytes activated with PHA without immune cell interaction displayed reduced expression of HLA-G and TNF-α, with no significant changes in IL-6, IFN-γ, IL-4, IL-10, VEGF, PDGF, PTGS-2, TGF-β, IDO, and iNOS compared to PHA-stimulated undifferentiated MSCs. Conclusion: This study demonstrates that chondrocytes derived from WJ-MSCs exhibit limited immunomodulatory potential compared to undifferentiated MSCs, particularly under PHA-induced inflammatory conditions. Undifferentiated MSCs showed superior regulation of key cytokines associated with immune modulation. These findings suggest that maintaining MSCs in an undifferentiated state may be advantageous for therapeutic applications targeting inflammatory conditions, such as osteoarthritis. Future research should explore strategies to enhance the immunomodulatory efficacy of chondrocytes, potentially through genetic modification or adjunctive therapies.

## 1. Introduction

Progressive articular cartilage damage is a significant global health issue that leads to dysfunctional joints, adversely affecting the quality of life and, in severe cases, becoming life-threatening [1]. Osteoarthritis (OA), the most prevalent joint disorder worldwide, is a chronic inflammatory condition characterized by progressive degradation of articular cartilage, synovial inflammation, and changes to subchondral bone. The inflammatory response in OA plays a critical role in cartilage degeneration, with immune cells and pro-inflammatory cytokines (e.g., IL-1β, TNF-α, and IL-6) contributing to matrix breakdown and chondrocyte apoptosis [2,3]. This inflammatory environment exacerbates joint damage and limits the efficacy of conventional treatments.

Conventional arthroscopic interventions for cartilage tissue injury, including partial meniscectomy, chondroplasty, mosaicplasty, synovectomy, removal of offending osteophytes, and microfracture, primarily offer palliative relief by alleviating knee pain and providing short-term benefits [4,5,6]. However, these procedures do not address the underlying degenerative processes responsible for cartilage degradation. In recent years, cell-based regenerative therapies, such as autologous chondrocyte implantation (ACI), have gained prominence as potential standard treatments for cartilage repair, with the expectation of offering long-term efficacy in reversing degenerative conditions [7,8]. Articular chondrocytes, the sole cell type found in cartilage, are sparsely distributed within the extracellular matrix (ECM) [9]. Under in vitro culture conditions, these chondrocytes exhibit limited proliferation potential, leading to the production of a poorly functioning and low-quality ECM [10]. Moreover, clinical applications of chondrocytes are hampered by factors such as donor tissue scarcity, donor site morbidity, chondrocyte dedifferentiation during culture, poor graft attachment to the surrounding chondral surface, and the inability to restore native tissue integrity [10]. These challenges often result in the formation of fibrocartilage, which is functionally inferior to the desired hyaline cartilage [11,12]. The role of arthroscopy in managing osteoarthritic knees has recently come under scrutiny due to inconsistent evidence regarding its effectiveness [13,14].

Mesenchymal stromal cells (MSCs) have emerged as promising candidates for cell-based regenerative therapies aimed at addressing progressive dysfunctional articular cartilage and the resultant degenerative loss of joint function [15,16,17,18]. MSCs are particularly relevant in the context of OA, as they not only have the potential to regenerate cartilage tissue by differentiation towards chondrocytes but also modulate inflammatory responses through paracrine signaling [19]. They also maintain hypoimmunogenicity, owing to low levels of major histocompatibility complex (MHC) and co-stimulatory proteins [19,20,21,22]. These characteristics enable MSCs to evade the recipient’s immune system, making them suitable for allogeneic transplantation. Numerous pre-clinical and clinical studies have demonstrated the safety and efficacy of undifferentiated MSCs in treating various immunological and autoimmune disorders, including OA [23]. Osteoarthritis is marked by an imbalance between pro- and anti-inflammatory cytokines, leading to chronic inflammation that impairs tissue repair [24]. MSCs can counteract these effects by secreting anti-inflammatory molecules such as IL-10, TGF-β, and indoleamine 2,3-dioxygenase (IDO), which suppress immune cell activity and create a regenerative environment [25,26]. These properties make MSCs attractive for treating inflammatory conditions like OA and rheumatoid arthritis (RA).

In our previous study, MSCs derived from the umbilical cord were co-cultured with immune cells under in vitro inflammatory conditions, resulting in a significant increase in the levels of specific cytokines, such as VEGF and IDO, at both gene and protein levels [27]. Building on these findings, the current study aims to investigate the immunomodulatory properties of chondrocytes differentiated from Wharton’s jelly-derived MSCs (WJ-MSCs), with a focus on their ability to regulate pro-inflammatory and anti-inflammatory markers in response to immune stimulation. Wharton’s jelly (WJ), a component of the umbilical cord, is an attractive source for MSCs due to its non-invasive collection, abundance, and secretion of growth factors that promote tissue regeneration. As a result, WJ-MSCs have gained significant attention as an allogeneic source for cell-based therapies.

This study is designed to evaluate and compare the inflammatory responses of chondrocytes differentiated from WJ-MSCs and undifferentiated WJ-MSCs by co-culturing them with immune cells and stimulating them with phytohemagglutinin (PHA), with unstimulated cells serving as controls. Specifically, we aim to analyze the differential expression of pro-inflammatory cytokines and anti-inflammatory markers to better understand the immunomodulatory roles of differentiated and undifferentiated cells.

Cytokine profiling in this study was customized based on previously reported literature, focusing on key pro-inflammatory and anti-inflammatory markers that are critical for immune modulation and cartilage regeneration. The selected cytokines were chosen due to their established roles in inflammatory processes, immune regulation, and tissue repair, particularly in the context of mesenchymal stem cell (MSC) interactions with immune cells and chondrocyte differentiation. This tailored approach allows for a comprehensive analysis of cytokine expression in response to immune stimulation, building on findings from previous studies [27]. Understanding these differences is essential for optimizing cell-based therapies for cartilage regeneration and managing inflammatory conditions associated with osteoarthritis (OA).

## 2. Materials and Methods

### 2.1. Protocol Registration Details

All protocols were approved by the Institutional Committee for Stem Cell Research (IC-SCR) of the Stem Cell Research Centre (SCRC), Government Stanley Hospital (GSH), Chennai (Registration No NAC-SCRT/79/20152002)/Proposal No 01/2017 and Institutional Ethical Committee (ECR/131/Inst/TN/2013/RR-16 dated 17/02/2017) of the Government Stanley Medical College and Hospital, Chennai. The samples used in this study were obtained in accordance with the Declaration of Helsinki Ethical Principles of Medical Research Involving Human Subjects. Thus, informed consent (IC) was obtained from the patients prior to sample collection.

### 2.2. Isolation of Mesenchymal Stem Cells from Wharton’s Jelly of Umbilical Cord by Explant Method

After obtaining informed consent from the mothers, human umbilical cords (Wharton’s jelly; WJ; *n* = 10) were aseptically collected from patients (mean age 26 ± 5 years) who underwent cesarean section deliveries at the Government Raja Sir Ramaswamy Mudaliar (RSRM) Hospital, an obstetrics section of Government Stanley Hospital (GSH), Chennai, India. The umbilical cords were collected in a sterile container containing transport medium maintained at 4 °C and processed within one hour by explant method as per our previous standardized protocol [28]. On day 7, explants were removed, and the medium was changed every 48–72 h thereafter. Upon reaching 80% confluence, the cells were trypsinized (0.25% trypsin–EDTA) and subsequently passaged at a cell density of 5000 cells/cm². MSCs isolated from the WJ source showed typical spindle morphology when observed under the microscope, which is one of the characteristic features of MSCs according to the minimal criteria proposed by ISCT [29].

### 2.3. Immunophenotyping of MSCs Derived from Wharton’s Jelly (WJ)

MSCs from the WJ source at passage 3 were characterized for MSC-specific markers as per the criteria defined by the International Society for Cellular Therapy (ISCT) [29]. Cells obtained from the WJ source were trypsinized and washed with 1× PBS. A cell density of 5 × 10^4^ cells/mL was stained with antibodies. Cells were stained with MSC-positive markers, including APC-conjugated anti-human CD73, FITC-conjugated anti-human CD90, and PerCP-Cy5.5-conjugated anti-human CD105, and negative markers, including FITC-conjugated anti-human CD14, CD45, and PE-conjugated anti-human CD34 antibodies (BD Biosciences, Franklin Lakes, NJ, USA). Stained cells were subjected to flow cytometry analysis using a multi-color flow cytometer (FACS ARIA II, Piscataway, NJ, USA) with FACS DIVA software (Version 6.1.2). Compensation was performed to reduce the spectral spillover of fluorochromes and mean fluorescent intensity was calculated. Events recording more than 10,000 were considered significant for calculating fluorescence intensity.

### 2.4. Differentiation Potential of MSCs Derived from Wharton’s Jelly

Tri-lineage differentiation was performed for the MSCs derived from all sources as per our established protocol mentioned in our previous publication [28]. MSCs from the WJ source at passage 3 were differentiated into osteogenic, adipogenic, and chondrogenic lineages using specific differentiation media. Tri-lineage differentiation was confirmed by Alizarin Red, Oil Red O, and Alcian Blue staining, respectively, to fulfill the minimal criteria proposed by the International Society for Cellular Therapy (ISCT) [29]. Further, chondrocyte differentiation was validated by chondrogenic-specific primers Sox9 and Col2A1 (type 2 collagen) and Col1A (type 1 collagen) as a dedifferentiation marker/fibroblast marker.

### 2.5. Co-Culturing of Chondrogenic Differentiated WJ-MSCs with MNCs in Non-Activated and PHA-Activated Conditions

WJ-MSCs (passage 3) were differentiated into chondrocytes using micro mass culture methods (five to six micro mass pellets were created in a single well) in six-well plates as per our established protocol [27]. After ensuring complete differentiation of MSCs into chondrocytes, mononuclear cells (MNCs) were seeded onto the differentiated chondrocytes. Phytohemagglutinin (PHA) was used for stimulation at a concentration of 10 µg/mL, and the co-culture system was incubated at 37 °C in 5% CO_2_ for 3 days. The various conditions of the study were grouped as follows:

Group 1: Chondrocytes alone (Control)

Group 2: PHA-stimulated chondrocytes (Chondrocytes + PHA)

Group 3: Chondrocytes co-cultured with MNCs (Chondrocytes + MNC)

Group 4: Chondrocytes co-cultured with activated MNCs (Chondrocytes + MNC + PHA)

The study performed on WJ-MSCs differentiated into chondrocytes was compared with similar co-cultured groups of undifferentiated WJ-MSCs to understand the cultural behavioral aspects of differentiated and undifferentiated cells under mitogen-stimulated conditions.

After 3 days, the MNCs in the suspension were removed. Morphological changes were examined using an inverted microscope. Cytokine profiling was performed using the RT^2^ Customized Gene Array. RNA extraction and custom RT^2^ profiler PCR array were performed as per our previous publication [27]. Changes in the expression of cytokines between the two groups were compared and analyzed (Table 1).

We used three biological replicates owing to the sensitivity of the experiments in which internal controls were used. Due to the highly sensitive nature of the assay and the presence of various groups in the experiment, our study design reflects a pilot scale rather than a clinical trial scale. Therefore, we obtained minimum biological replicates (*n* = 3 different samples) with technical duplicates to allow for generating statistical power in our inference.

### 2.6. Quantitative Protein Expression Studies by Solid-Phase Sandwich Enzyme-Linked Immunosorbent Assay (ELISA)

In addition to gene expression, protein expression for undifferentiated MSCs derived from Wharton’s jelly and chondro-differentiated cells of the co-cultured groups mentioned in Section 2.4 was assessed by solid-phase sandwich ELISA. Briefly, MSCs and chondro-differentiated cells were harvested after 3 days of co-culture, and the cell pellet was lysed using RIPA buffer supplemented with a protease inhibitor cocktail (10 µg/mL). To ensure complete lysis of the cell pellet, sonication was carried out by pulsing for 10 s at a frequency of 20 kHz. The cell lysate was then centrifuged at 4 °C for 45 min at 14,000 rpm, and the supernatants were collected. The total protein concentration was determined by the Bradford protein estimation method. A total protein amount of 50 µg was used to determine the expression of angiogenic growth factor VEGF. The concentration of VEGF was quantified by ELISA following the manufacturer’s protocol (Human VEGF-A ELISA Kit—Cat No. BMS277, Invitrogen, Carlsbad, CA, USA).

Briefly, the cell lysate of all samples (50 µg of total protein diluted in the diluent provided in the kit) and standards were added to the VEGF antibody pre-coated wells and incubated for 2.5 h at 37 °C. This was followed by incubation for one hour with a biotin conjugate, which binds to the primary antibody. Subsequently, a secondary antibody, streptavidin-HRP conjugate, which binds with the biotin conjugate, was added and incubated for 45 min. Unbound antibodies were removed by washing at each step. Finally, TMB substrate solution was added to the plates, incubated for 30 min, and observed for color development to determine the amount of protein present in the sample. The reaction was terminated by the addition of a stop solution, and absorbance was recorded at 450 nm using an ELISA reader (Robonik Readwell, Mahape Navi Mumbai, Maharashtra, India). The concentration of VEGF proteins was calculated using their respective standards. Finally, results were expressed as protein expression (VEGF) in pg per milligram of total protein.

### 2.7. Statistical Analysis

All experiments were performed in biological triplicates (*n* = 3). Statistical analysis was performed using one-way ANOVA and multiple t-tests with GraphPad Prism version 10.4 for Windows (GraphPad Software, La Jolla, CA, USA). For all statistical analyses, *p* < 0.05 was considered significant (* *p* < 0.05, ** *p* < 0.01, *** *p* < 0.001, and **** *p* < 0.0001).

## 3. Results

### 3.1. Isolation and Morphological Characterization

**Figure 1:**
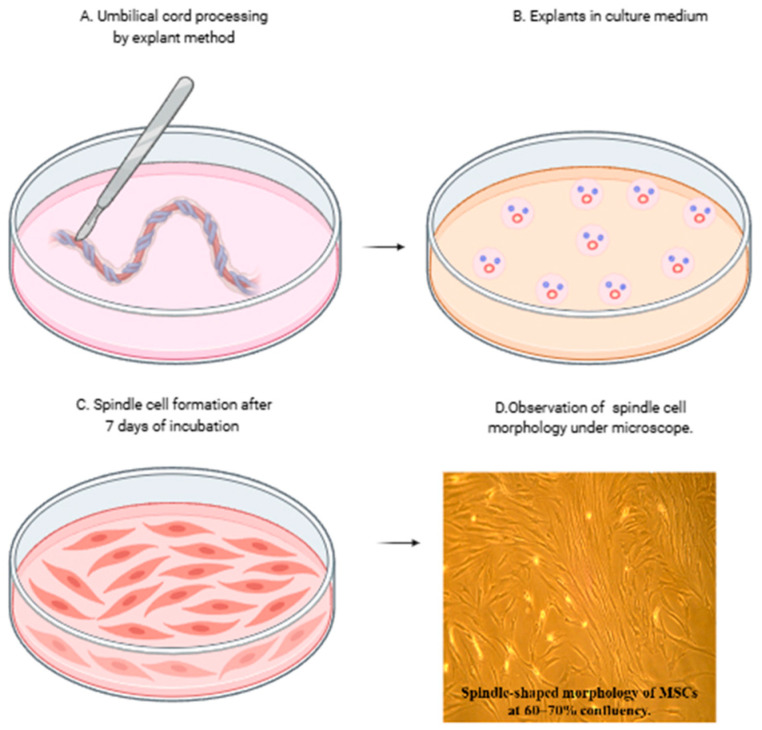
Isolation and morphological characteristics of mesenchymal stromal cells (MSCs) from umbilical cord. (**A**,**B**) Isolation of mesenchymal stromal cells from the umbilical cord by explant method and (**C**) spindle cell formation after removal of explants at day 7. (**D**) MSCs showing characteristic spindle-shaped morphology under the phase contrast microscope at 10× magnification.

MSCs were successfully isolated from Wharton’s jelly and exhibited adherence to tissue culture plastic. The cells displayed a characteristic spindle-shaped fibroblast-like morphology under phase-contrast microscopy (Figure 1).

### 3.2. Immunophenotypic Analysis

**Figure 2:**
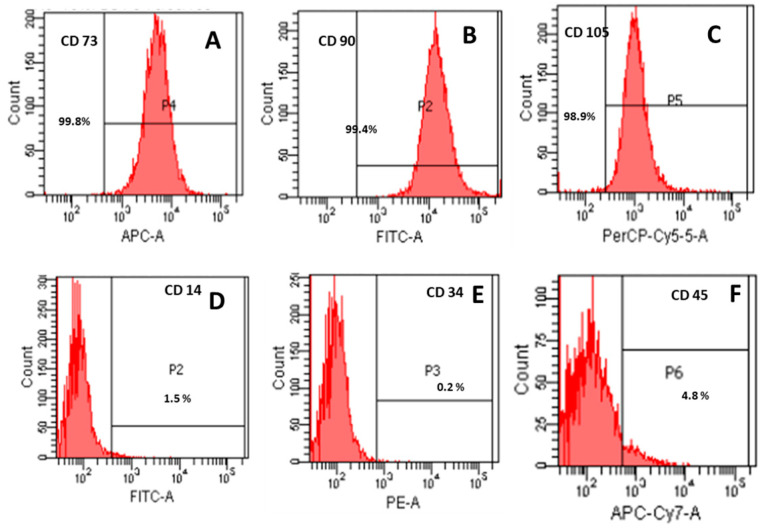
Immunophenotypic characterization of mesenchymal stromal cells (MSCs) according to International Society for Cellular Therapy (ISCT) criteria. Mesenchymal stromal cells for the positive expression of CD 73, CD 90, and CD 105 (**A**–**C**) and lack of expression of CD14, CD34, and CD45 (**D**–**F**).

Flow cytometric analysis confirmed the expression of mesenchymal stem cell surface markers CD73 (99.8%), CD90 (99.4%), and CD105 (98.9%), while hematopoietic markers such as CD14 (1.5%), CD34 (0.2%), and CD45 (4.8%) were absent (<5%), consistent with ISCT criteria for MSCs (Figure 2).

### 3.3. Tri-Lineage Differentiation

**Figure 3:**
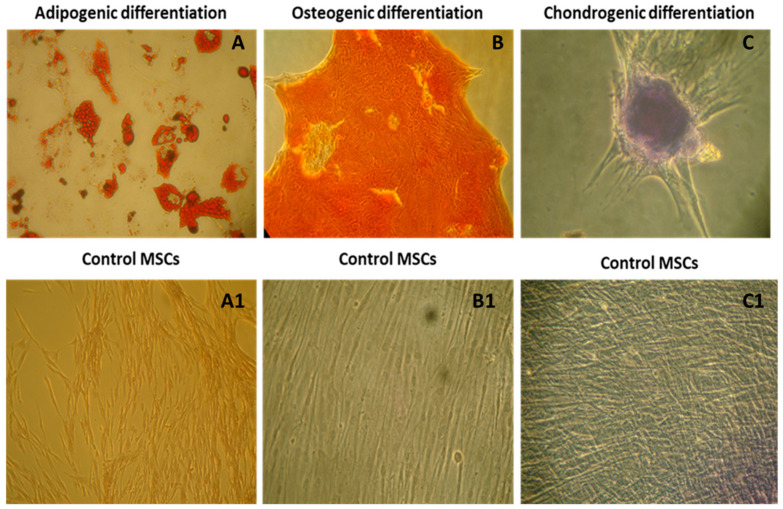
Characterization of mesenchymal stromal cells by tri-lineage differentiation according to the minimal criteria proposed by ISCT. (**A**–**C**) Confirmation of adipogenic differentiation by Oil-Red O staining, osteogenic differentiation by Alizarin Red staining, and chondrogenic differentiation by Alcian Blue staining with their respective controls (**A1**–**C1**). All the images were taken at 10× magnification under a phase contrast microscope.

The multipotent ability of WJ-MSCs was validated through differentiation into adipogenic, osteogenic, and chondrogenic lineages under specific induction conditions. Adipogenic differentiation was confirmed by Oil Red O staining for lipid droplets, osteogenic differentiation by Alizarin Red staining for mineral deposits, and chondrogenic differentiation by Alcian Blue staining for glycosaminoglycans (Figure 3).

### 3.4. Chondrogenic Differentiation and Gene Expression

**Figure 4:**
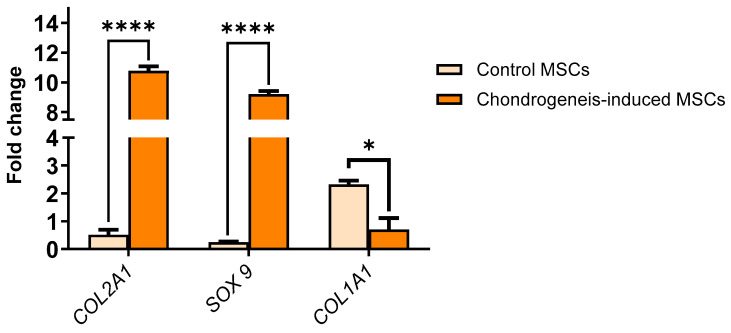
Relative gene expression levels of chondrocyte-specific markers (SOX9 and COL2A1) and fibroblast-associated marker (COL1A1) in MSCs after chondrogenic differentiation. SOX9 and COL2A1 were significantly upregulated in differentiated cells compared to undifferentiated controls), confirming successful chondrogenic differentiation (**** *p* < 0.0001). Expression of COL1A1 was negligible, indicating the absence of fibroblast contamination (* *p* < 0.05). Data are presented as mean ± SEM (*n* = 3 biological replicates), normalized to GAPDH expression. Statistical significance was determined using multiple *t*-test analysis.

Chondrogenic differentiation was further validated by the upregulation of chondrocyte-specific markers. Quantitative PCR demonstrated increased expression of SOX9 and COL2A1 in differentiated cells compared to undifferentiated controls (fold change COL2A1, 10.7: SOX9, 9.2, *** *p* < 0.001) (Figure 4). The expression of fibroblast-associated COL1A1 was negligible in differentiated cells, confirming the specific differentiation into mature chondrocytes.

### 3.5. Influence of PHA Activation on Morphological Changes During Co-Culture of WJ-MSC-Derived Chondrocytes with MNCs

The morphological changes in chondrocytes based on size were measured across all groups, including PHA-activated chondrocytes, co-cultured chondrocytes with and without PHA stimulation, and control unstimulated chondrocytes. After 3 days of co-culture, drastic changes in the morphology of chondrocytes were observed among different groups. The sizes of chondrocytes under various conditions are as follows: (i) chondrocytes: 140 ± 0.5 µm, (ii) chondrocytes + PHA: 697 ± 0.5 µm, (iii) chondrocytes + MNC: 202 ± 0.5 µm, and (iv) chondrocytes + MNC + PHA: 450 ± 0.5 µm.

**Figure 5:**
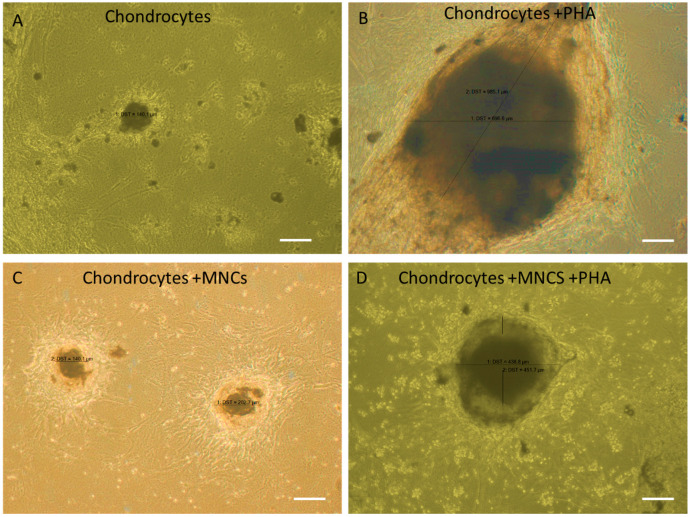
Influence of PHA activation on the morphological changes during co-culture of WJ-MSC-derived chondrocytes with MNC. (**A**) Micrograph of chondrocytes showing the formation of micro-mass pellet after 21 days of chondrogenic differentiation (negative control). (**B**) Chondrocytes treated with PHA (positive control) showing an increase in size, indicating responsiveness to PHA. (**C**) Chondrocytes co-cultured with MNCs in the absence of PHA, showing no difference in their morphology. (**D**) Chondrocytes co-cultured with immune cells in the presence of PHA, showing a decrease in cell size compared to positive control, indicating responsiveness towards PHA. Cell size measured by Image J software (latest v. 1.54).

When chondrocytes were stimulated with PHA, their size increased nearly threefold (697 µm) compared to unstimulated chondrocytes (140 µm), indicating responsiveness towards the mitogen PHA. When immune cells were added to this inflammatory microenvironment, the size of chondrocytes was reduced by approximately one-fourth (450 µm), indicating cross-talk between chondrocytes and immune cells under inflammatory conditions. However, when chondrocytes were co-cultured with immune cells in the absence of PHA, there was no significant change, but a slight increase in size compared to control chondrocytes and a decrease in size (reduced to nearly half) compared to the activated co-culture condition. This indicates some responsiveness of chondrocytes towards immune cells. These results suggest that PHA plays a significant role in the observed morphological changes by influencing the increase in size. Consequently, changes in the gene as well as protein expression levels of pro- and anti-inflammatory cytokines were expected. Surprisingly, changes in gene expression were observed in non-activated co-cultured chondrocytes more than in activated co-cultured chondrocytes (Figure 5).

### 3.6. Expression of Pro- and Anti-Inflammatory Cytokines When Chondrocytes Crosstalk with Immune Cells Under PHA Stimulation

**Figure 6:**
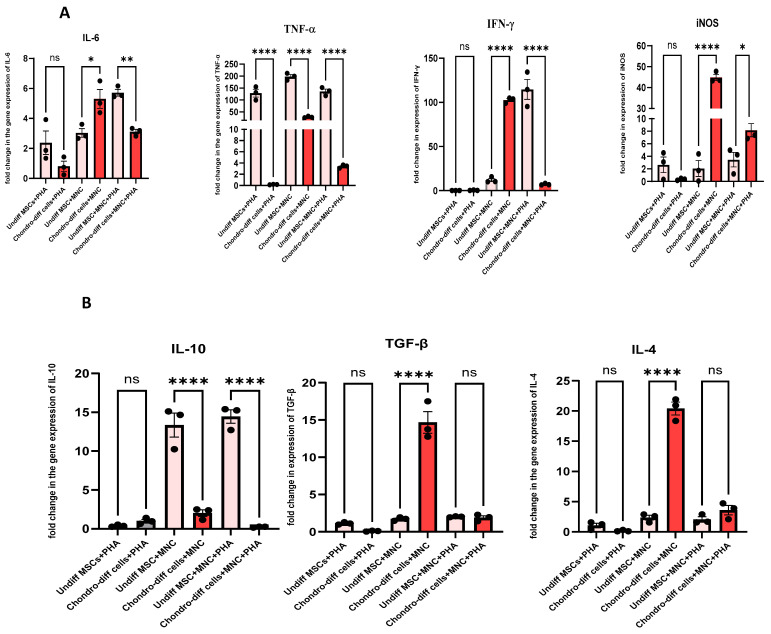

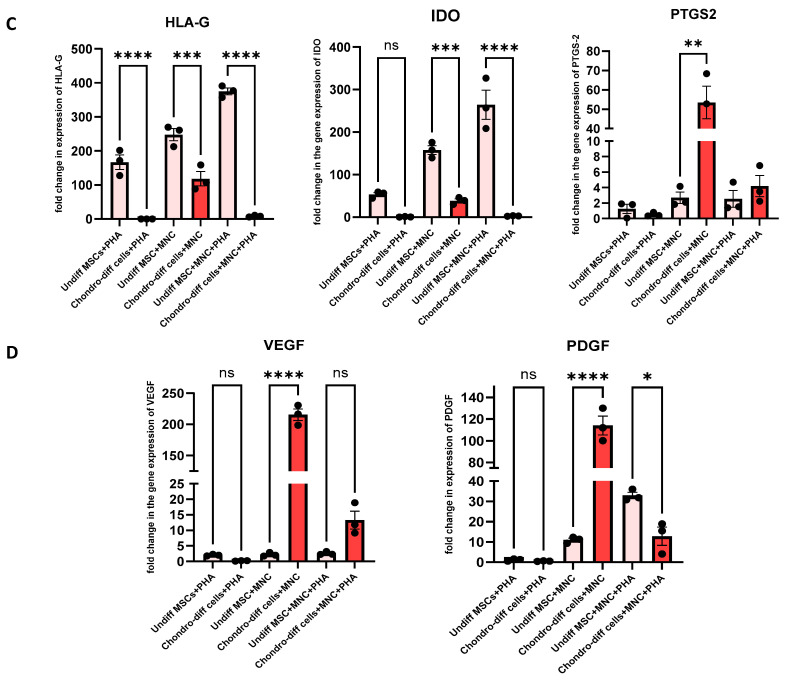
(**A**) Pro-inflammatory Cytokines: Expression levels of IL-6, TNF-α, and IFN-γ,iNOS were compared under three conditions: co-culture of chondrocytes with immune cells under PHA stimulation, co-culture of chondrocytes without PHA stimulation, and PHA stimulation alone. Significant downregulation of IL-6, IFN-γ, and TNF-α was observed in the PHA-activated co-culture condition compared to other conditions (* *p* < 0.05, ** *p* < 0.0, *** *p* < 0.001, and **** *p* < 0.0001). (**B**) Anti-inflammatory cytokines: Expression levels of IL-10, TGF-β, and IL-4 were compared under three conditions: co-culture of chondrocytes with immune cells under PHA stimulation, co-culture of chondrocytes without PHA stimulation, and PHA stimulation alone. Significant downregulation in the level of IL-10, with no significant changes in the level of IL-4 and TGF-β was observed in the PHA-activated co-culture condition in differentiated chondrocytes. (**C**) Immunomodulatory molecules: HLA-G and IDO expression was reduced in differentiated chondrocytes across all conditions compared to undifferentiated MSCs. (**D**) Growth factors: VEGF and PDGF were significantly upregulated in non-PHA co-cultured conditions. Data were analyzed using GraphPad Prism 10.4 with one-way ANOVA and Sidak’s multiple comparisons test. Values are presented as mean ± SEM.

When chondrocytes derived from WJ-MSCs were co-cultured with immune cells under PHA stimulation (activated co-culture conditions), the expression of key pro-inflammatory cytokines, including IL-6, TNF-α, and IFN-γ was significantly downregulated compared to undifferentiated MSCs under similar conditions (Figure 6A). This suggests that while chondrocytes retain some capacity to suppress pro-inflammatory stimuli, their immunomodulatory efficacy is reduced compared to undifferentiated MSCs. For anti-inflammatory cytokines, such as IL-10 and TGF-β, the expression was also downregulated in chondrocytes compared to MSCs, indicating a diminished ability to enhance anti-inflammatory responses under inflammatory conditions (Figure 6B). VEGF, an angiogenic factor critical for tissue repair, and checkpoint molecules like HLA-G and IDO were also reduced in chondrocytes in these conditions, further highlighting the limited immunosuppressive capability of differentiated cells (Figure 6C,D).

In contrast, under non-PHA co-cultured conditions, the expression of certain anti-inflammatory cytokines, including IL-4, PTGS-2, PDGF, iNOS, VEGF, and TGF-β, were upregulated in chondrocytes (Figure 6A–D). These findings suggest that in the absence of strong inflammatory stimulation, chondrocytes maintain some ability to promote a reparative and anti-inflammatory environment. However, pro-inflammatory markers such as IL-6 and iNOS were also upregulated in these conditions, indicating a mixed response. Notably, TNF-α was downregulated in these conditions, suggesting a partial suppression of inflammation (Figure 6A). HLA-G and IDO were consistently downregulated in chondrocytes compared to undifferentiated MSCs, regardless of the co-culture or PHA stimulation status, indicating that these key immunosuppressive molecules are largely lost during differentiation into chondrocytes (Figure 6C).

When chondrocytes were activated with PHA without immune cell interaction, there were no significant changes in most pro- and anti-inflammatory cytokines compared to PHA-stimulated undifferentiated MSCs. Only TNF-α and HLA-G showed downregulation, while cytokines such as IL-6, PTGS-2, TGF-β, IDO, IFN-γ, IL-4, IL-10, VEGF, PDGF, and iNOS remained unchanged (Figure 6A–D). This unresponsiveness to PHA stimulation highlights the limited ability of chondrocytes to mount a significant pro- or anti-inflammatory response without the presence of immune cells, further differentiating their behavior from undifferentiated MSCs.

### 3.7. ELISA Quantification of Angiogenic Growth Factor VEGF

**Figure 7:**
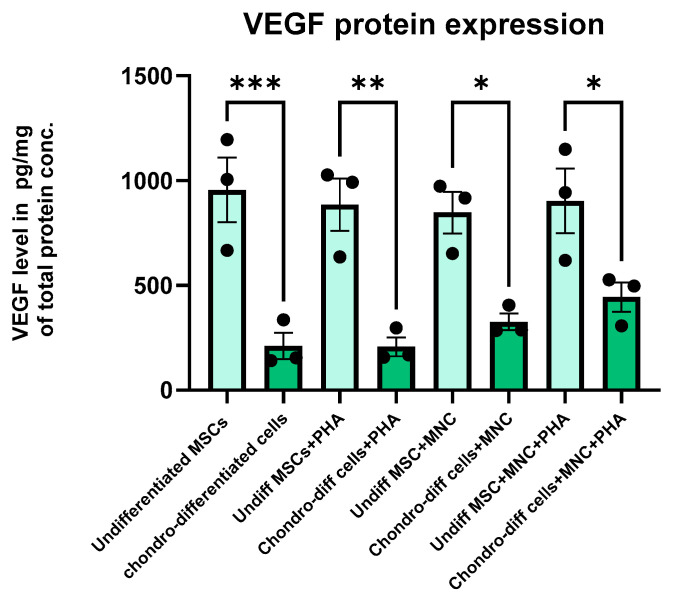
Bar graph showing the quantitative intracellular protein levels by solid-phase sandwich ELISA for VEGF during the various culture conditions. VEGF expression was significantly higher in undifferentiated MSCs compared to chondro-differentiated cells in all the culture conditions (* *p* < 0.05, ** *p* < 0.01, *** *p* < 0.001) Data were analyzed using GraphPad Prism 10.4 with one-way ANOVA and Sidak’s multiple comparisons test. Values are presented as mean ± SEM.

Protein expression levels of VEGF were quantified using solid-phase sandwich ELISA. The results demonstrated that undifferentiated WJ-MSCs exhibited higher levels of VEGF compared to their chondro-differentiated counterparts in all the culture conditions. Among chondro-differentiated cells, VEGF expression was significantly elevated in co-cultured chondrocytes (both non-activated and PHA-activated) than the chondrocytes alone and PHA-activated chondrocytes, aligning with the gene expression data (Figure 7) indicating the secretion of VEGF by chondrocytes during interaction with immune cells.

## 4. Discussion

This study aimed to delineate the interaction between chondrocytes differentiated from Wharton’s jelly-derived mesenchymal stromal cells (WJ-MSCs) and immune cells under phytohemagglutinin (PHA)-stimulated conditions, comparing these interactions with those of undifferentiated MSCs. The primary objective was to understand the immunomodulatory differences between differentiated and undifferentiated cells, which is crucial for optimizing cell-based therapies for cartilage regeneration and managing inflammatory conditions associated with osteoarthritis (OA).

Lohan et al. reported that rat chondrocytes cultured under normoxic or hypoxic conditions followed by in vitro co-culture tests with allogeneic lymphocytes and macrophages demonstrated the lack of obvious immunogenicity, despite exposure to a pro-inflammatory environment, combined with the immunomodulatory ability. Thus, they suggested that these cells have the ability to evade the host immune system and suppress inflammation, potentially facilitating the resolution of OA-induced inflammation and cartilage regeneration [30]. We performed a similar study utilizing chondrocytes differentiated from MSCs and undifferentiated MSCs co-cultured with lymphocyte cultures under PHA-stimulated conditions. Our findings indicate that undifferentiated WJ-MSCs possess superior immunomodulatory potential compared to their chondro-differentiated counterparts. This is contradictory to the above study since chondrocytes displayed both anti-immunogenic and immunoregulatory properties. In our study specifically, pro-inflammatory cytokine levels (IL-6, TNF-α, and IFN-γ) were significantly downregulated in co-cultured chondrocytes, whereas only a few anti-inflammatory cytokines (IL-10, TGF-β, IL-4, and VEGF) were upregulated, and only in the absence of PHA stimulation [31,32]. This selective modulation suggests that chondrocytes retain some immunosuppressive capabilities but lack the robust anti-inflammatory response observed in undifferentiated MSCs. Thus, MSCs from Wharton’s jelly source harboring novel tropic signaling factors ameliorating the inflamed milieu similar to the native chondrocyte could be potentially considered for therapeutic applications.

The absence of HLA-G expression and reduced IDO levels in differentiated chondrocytes, as opposed to undifferentiated MSCs, underscores a potential shift in the immunomodulatory profile upon differentiation [33]. HLA-G is a known checkpoint molecule that confers protection against the immune system during transplantation [31]. Its absence in chondrocyte-differentiated cells could imply increased immunogenicity, although this was not directly assessed in our study. Similarly, IDO, an enzyme involved in tryptophan metabolism with immunosuppressive functions, was downregulated in differentiated chondrocytes, suggesting a diminished capacity for immune modulation [32]. Contrary to our expectations, PHA stimulation did not significantly alter the expression of pro- or anti-inflammatory cytokines in chondrocytes, similar to undifferentiated MSCs. This unresponsiveness to PHA suggests that the immunomodulatory capacity may be influenced by the cellular source or the differentiation process itself. Previous studies have reported conflicting results regarding the immunomodulatory properties of MSCs post-differentiation [34,35,36,37]. For instance, Chen et al. [38] demonstrated that chondrogenic differentiation alters the immunosuppressive properties of bone marrow-derived MSCs by upregulating B7 molecules, thereby increasing immunogenicity. In contrast, Voisin et al. [39]. reported that WJ-MSCs retained their hypo-immunogenic and immunomodulatory properties even after chondrogenic differentiation. These discrepancies highlight the complexity of MSC biology and the need for standardized differentiation protocols and immunomodulatory assessments.

The reduced upregulation of anti-inflammatory cytokines in differentiated chondrocytes compared to undifferentiated MSCs suggests a potential limitation in the therapeutic efficacy of chondrocyte-based therapies, especially in inflammatory environments characteristic of OA [40]. While chondrocytes can suppress pro-inflammatory cytokines to some extent, their limited ability to enhance anti-inflammatory cytokines may impede their capacity to create a conducive environment for cartilage regeneration and healing [41]. Our morphological analysis revealed that PHA stimulation significantly increased the size of chondrocytes, indicating cellular activation. However, the subsequent addition of immune cells reduced this size, suggesting cellular interactions that may modulate the overall response. The morphological changes, coupled with the cytokine expression profiles, indicate that while chondrocytes respond to inflammatory stimuli, their immunomodulatory response is not as comprehensive as that of undifferentiated MSCs.

VEGF are key regulators of angiogenesis responsible for tissue repair and regeneration after injury [42,43]. They play a major role in immunity as well as inflammation. During inflammation, VEGF is responsible for the recruitment of inflammatory cells and the expression of co-stimulatory molecules on recruited and resident monocytes, leading to the upregulation of pro-inflammatory cytokine expression at the injured site [44]. VEGF acts on dendritic cells (DCs) to produce IDO, an immunosuppressive molecule that inhibits the further proliferation and activation of DCs and T cells and induces T cell apoptosis [45]. Under the stimulation of pro-inflammatory cytokines at the site of injury, immigrated MSCs secrete a plethora of growth factors, including VEGF, EGF, PDGF, FGF, etc. [46]. VEGF plays an important role in tissue repair and regeneration by enhancing angiogenesis, eliciting progenitor cell differentiation, and inhibiting leukocyte transmigration [45]. VEGF plays a pivotal role in angiogenesis and tissue regeneration, and its higher expression in MSCs suggests a more robust regenerative potential [47,48,49].

In chondrocytes, the VEGF protein expression analysis via ELISA corroborated the gene expression findings, indicating a consistent relationship between transcriptional activity and protein production. In contrast, the low VEGF gene expression but high VEGF protein levels observed in undifferentiated cells suggest the involvement of post-transcriptional regulation or protein stability mechanisms that enhance VEGF protein abundance despite low mRNA levels. Additionally, VEGF protein accumulation in the microenvironment, autocrine or paracrine feedback, and differential regulation of transcription and post-translational processes may contribute to this discrepancy.

The implications of these findings are complex. On one hand, the retention of some immunosuppressive capabilities in chondrocytes post-differentiation supports their use in cell-based therapies aimed at reducing inflammation. On the other hand, the diminished expression of key immunomodulatory molecules like HLA-G and IDO raises concerns about the long-term efficacy and safety of chondrocyte-based therapies, particularly in immunocompetent hosts. Future studies should focus on elucidating the mechanisms underlying the altered immunomodulatory profiles of differentiated chondrocytes. Investigating the signaling pathways and transcriptional networks involved in MSC differentiation and their subsequent interaction with immune cells could provide deeper insights [50,51,52]. Additionally, expanding the study to include a broader range of cytokines and immune markers, as well as assessing functional outcomes in relevant animal models, would enhance the understanding of chondrocyte immunomodulation in vivo. Exploring strategies to enhance the immunomodulatory capacity of chondrocytes, such as genetic modification or co-delivery with immunomodulatory agents, could improve their therapeutic potential [53]. Comparative studies involving MSCs from different sources and standardized differentiation protocols would also help in reconciling the conflicting reports in the literature.

## 5. Conclusions

Chondrocytes derived from WJ-MSCs exhibit limited immunomodulatory activity compared to undifferentiated MSCs. The differentiated chondrocytes showed downregulation of key pro-inflammatory cytokines (IL-6, TNF-α, and IFN-γ) but only modest upregulation of anti-inflammatory cytokines (IL-10, TGF-β, and IL-4) and growth factors (PDGF and VEGF) in non-PHA conditions. Crucially, key immunomodulatory molecules like HLA-G and IDO were reduced, highlighting diminished immune modulation post-differentiation. Morphological analysis also indicated that PHA significantly altered cell size, suggesting activation, but subsequent immune cell interaction reduced this effect. Overall, the diminished anti-inflammatory response and lack of significant modulation with PHA suggest that undifferentiated MSCs retain superior therapeutic potential, particularly for inflammatory conditions. Future optimization strategies might include genetic enhancement of chondrocytes or co-delivery with immunomodulatory agents. These findings suggest that maintaining MSCs in their undifferentiated state is preferable for robust immune modulation in osteoarthritis therapies.

## Figures and Tables

**Table 1:** Cytokines customized for gene array.

Sl. No.	Cytokines	Gene Symbol	Gene Bank
1	Prostaglandin-endoperoxide synthase 2	PTGS2	NM_000963
2	Transforming growth factor-beta 2	TGFB2	NM_003238
3	Interferon, gamma	IFNG	NM_000619
4	Interleukin 10	IL-10	NM_000572
5	Interleukin 6 (interferon, beta 2)	IL-6	NM_000600
6	Interleukin 4	IL-4	NM_000589
7	Platelet-derived growth factor-beta polypeptide	PDGFB	NM_002608
8	Human leukocyte antigen G	HLA-G	NM_002127
9	Indolamine 2,3 dioxygenase 1	IDO	NM_002164
10	Vascular endothelial growth factor	VEGF	NM_001025366
11	Tumor necrosis factor	TNF-α	NM_000594
12	Nitric oxide synthase 2, inducible	NOS2	NM_000625
13	B-actin	ACTB	NM_001101
14	Human genomic DNA contamination	HGDC	Control
15	Positive PCR control	PPC	Control
16	Reverse transcription control	RTC	Control

## Data Availability

The datasets generated and analyzed during the current study are available from the corresponding author (J.S.) upon reasonable request.
